# Scaling up tuberculosis preventive treatment in Brazil: the ExpandTPT way forward

**DOI:** 10.36416/1806-3756/e20250154

**Published:** 2025-07-22

**Authors:** Anete Trajman, Ezio Távora dos Santos, Ricardo Arcêncio

**Affiliations:** 1. Disciplina de Clínica Médica, Faculdade de Medicina, Universidade Federal do Rio de Janeiro, Rio de Janeiro (RJ) Brasil.; 2. Rede Brasileira de Pesquisas em Tuberculose - REDE-TB - Universidade Federal do Rio de Janeiro, Rio de Janeiro (RJ) Brasil.; 3. Departamento de Enfermagem Materno-Infantil e Saúde Pública, Escola de Enfermagem de Ribeirão Preto, Universidade de São Paulo, Ribeirão Preto (SP) Brasil.

## TO THE EDITOR:

Tuberculosis elimination will not be possible without scaling up prevention through tuberculosis preventive treatment (TPT).^1^ ^,^ ^2^ Contacts of persons with pulmonary tuberculosis and people living with HIV/AIDS are the two largest eligible populations for TPT. Although the individual absolute risk of progression from infection to disease is higher among people living with HIV/AIDS,^3^ most new tuberculosis diagnoses are in contacts, who constitute the largest population in terms of attributable risk, especially in high-burden countries. However, there has been little progress in providing TPT to contacts worldwide,^4^ and Brazil is no exception.^5^

Contacts were, therefore, the target audience of the *Rede Brasileira de Pesquisas em Tuberculose* (REDE-TB, Brazilian Tuberculosis Research Network) ExpandTPT project, which was initially funded by the Stop TB Partnership (TB REACH Grant no. 10429) in five Brazilian capitals with a high tuberculosis burden (Manaus, Porto Alegre, Recife, Rio de Janeiro, and São Paulo; phase 1) and then extended to three other cities (João Pessoa, Nova Iguaçu, and Salvador; phase 2) with resources from the Brazilian National *Ministério da Ciência, Tecnologia e Inovação*, *Conselho Nacional de Desenvolvimento Científico e Tecnológico* (CNPq, National Council for Scientific and Technological Development; Grant no. 445684/2023-2). 

Phase 1 of the ExpandTPT project was conducted between April of 2023 and July of 2024 and was based on the lessons learned from the ACT4 cluster randomized clinical trial, which identified bottlenecks of the cascade of care for tuberculosis contacts and implemented tailored solutions.^6^ ^,^ ^7^ The ExpandTPT project was conducted in close partnership with the Brazilian National Tuberculosis Program, with participation of civil society advocates (through a community advisory board) and city-level tuberculosis programs. In brief, during technical visits with Brazilian National Tuberculosis Program officials, the ExpandTPT team, and the community advisory board, city tuberculosis program officials discussed the main bottlenecks in primary health care services, as well as customized solutions to improve the cascade of care for tuberculosis contacts. Those visits included meetings with municipal health department officials and managers of the services providing care to tuberculosis contacts. In addition to service reorganization, the main bottlenecks identified were the knowledge and skills required to perform the tuberculin skin test (TST). Therefore, more than 15,000 health professionals and community health workers received online training on the Brazilian national TST guidelines, the training course being provided by the ExpandTPT team and Brazilian National Tuberculosis Program officials. In 327 clinics where there was at least one new person with tuberculosis per month on average, ExpandTPT offered a simplified training course on the TST, the number of clinics offering the TST thus being expanded from 63 to 234 between October of 2023 and July of 2024. In addition, ExpandTPT provided in-service training using a dedicated contact registry book. The delay in training on the TST was due to a shortage of PPD RT23 in Brazil during the first six months of the project. 

A strategic component of the ExpandTPT project was the direct participation of a select group of tuberculosis advocates (the community advisory board) in each city. They monitored the training; contributed to its improvement and the planning of actions; developed specific materials for community health workers; participated in monitoring and technical visits; and proposed specific solutions to the city tuberculosis programs. 

**Figure 1:**
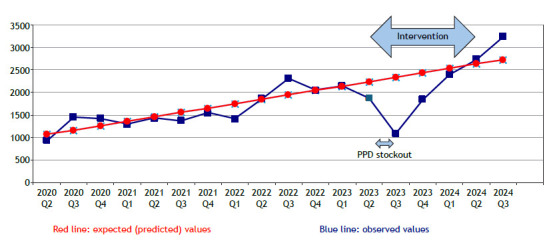
Number of tuberculosis preventive treatments prescribed to contacts reported in five cities in Brazil.

With the implementation of the actions, the overall number of TPTs prescribed to contacts in those five cities increased by 70% after a sharp drop in the first semester related to the lack of PPD, surpassing the expected values had no intervention occurred (Figure 1). This increase in the number of TPTs was due to an improvement in two steps of the cascade of care for tuberculosis contacts: an increase in the number of contacts identified, indicating the central role of community health workers; and, after TST training, an increase in the proportion of contacts tested (data not shown), showing the impact of the PPD stockout and revealing the fragility of depending on a single consumable, as well as the need to incorporate other technologies for the diagnosis of *Mycobacterium tuberculosis* infection. 

The ExpandTPT legacy also includes the following: a partnership among federal, state-level, and city-level programs to improve tuberculosis care in primary health care settings; visibility of civil society advocates and an understanding of the positive impact of their efforts on tuberculosis care; and simplification of recommendations for TST training,^8^ as a result of ExpandTPT advocacy activities and on the basis of evidence generated by ExpandTPT,^9^ as well as other evidence. 

A final ExpandTPT seminar was held in Brasilia on March 13, 2025, when the project partners agreed on the next steps. First, investments will be needed in order to leverage TPT in the country. Funding is needed for at least one nurse dedicated to TPT follow-up activities in city-level tuberculosis programs; for indirect costs of personnel time for training activities; and for community-based civil society activities. Resources are also needed for effective implementation of an industrial park that guarantees autonomy in the development and production of strategic consumables for the country. In the short term, we recommend prompt evaluation and incorporation of new technologies for the diagnosis of *M. tuberculosis* infection. Tuberculosis-specific skin tests are more cost-effective than the tests currently used in Brazil and would be an attractive alternative. Other rapid point-of-care blood-based tests are in the pipeline^10^ and should be evaluated for incorporation into the Brazilian public health system. 

Outcomes revealed how collaboration among city-level tuberculosis programs and their local partners is key to ensure rapid access to chest X-rays to accelerate and improve access to TPT. Expanding the network for tuberculin skin testing also proved to be pivotal in this effort, requiring increasing the number of trained professionals and ensuring that refrigerators are available for adequate PPD storage. 

Finally, social determinants were identified in the challenges on reception, testing, and follow-up at the primary health care level, as well as reaching out to contacts, revealing the need for restructuring services in a decentralized, accessible, and reliable logic under the view of service users.^11^

In conclusion, ExpandTPT leveraged TPT scale-up, but there is a need for improvement. The efforts and resources utilized to promote TPT expansion need to be guaranteed to further reach out and treat eligible contacts. 
